# The Role of Nesprin-4 in Breast Cancer Migration and Invasion

**DOI:** 10.3390/cells14191484

**Published:** 2025-09-23

**Authors:** Badria Fouad Al-Sammak, Lutfiye Yildiz Ozer, Hend Salah Fayed, Nada Mohamed Kafour, Johan Ericsson, Ayman Al Haj Zen, Henning F. Horn

**Affiliations:** College of Health and Life Sciences, Hamad Bin Khalifa University, Doha P.O. Box 34110, Qatar; baal32317@hbku.edu.qa (B.F.A.-S.); lutyildiz@gmail.com (L.Y.O.); hfayed@hbku.edu.qa (H.S.F.); nkafour@hbku.edu.qa (N.M.K.); pericsson@hbku.edu.qa (J.E.); aalhajzen@hbku.edu.qa (A.A.H.Z.)

**Keywords:** breast cancer, migration, invasion, intravasation, LINC complex, nesprin-4

## Abstract

Cancer metastasis is responsible for most cancer-related deaths. Migration and invasion, key steps in the metastatic cascade, require nuclear pliability to traverse the physical barriers of the extracellular matrix and cell–cell junctions. The nuclear envelope (NE) contains LINC complex proteins, including nesprin-4, which regulate nuclear integrity, stiffness, and cell movement. We report that nesprin-4 expression is generally upregulated in breast cancer samples but is reduced in triple-negative breast cancer (TNBC) samples compared to other subtypes. A nesprin-4 expression analysis in 62 breast cancer cell lines showed that nesprin-4 expression correlates positively with cell lines representing less aggressive tumors, while TNBC cell lines have low or no nesprin-4 expression. To determine the role of nesprin-4, we modulated nesprin-4 expression levels in three breast cancer cell lines: MCF7, T47D (luminal A and nesprin-4-positive), and MDA-MB-231 (TNBC and nesprin-4-negative). We found that nesprin-4 promotes migration and invasion by driving cell polarization. However, we also found that nesprin-4 impedes intravasation into endothelial microvessels. Thus, we propose that nesprin-4 plays a dual role in breast cancer, promoting efficient migration and invasion, but blocking intravasation.

## 1. Introduction

Breast cancer (BC) is the leading cause of cancer-related mortality, with most deaths resulting from metastasis [1,2,3,4,5]. Metastasis is a hallmark of cancer composed of a distinct progression of events that include invading surrounding tissues, intravasating blood vessels or lymphatic ducts, extravasating into new tissues, and establishing secondary tumors [5,6]. The metastatic process is associated with prerequisite changes in cell shape and behavior, many of which are associated with EMT (epithelial-to-mesenchymal transition), a developmental process subverted by cancer cells to promote a more invasive phenotype (recently reviewed in [7]).

Several steps in the metastatic cascade are mechanically demanding processes. These steps require dramatic cellular deformations as cells push through dense extracellular matrices (ECM) and confined interstitial spaces [8]. The cell nucleus is the largest and stiffest organelle and, as such, is a key bottleneck for successful cellular migration in restrictive spaces [8,9,10,11,12,13]. The mechanical attributes of the nucleus are largely determined by nuclear lamins and chromatin [14,15,16,17,18,19,20].

Lamins assemble into the nuclear lamina, a dense network of intermediate filaments beneath the inner nuclear membrane [21]. One of the functions of the nuclear lamina is to protect chromatin against mechanical stress and DNA damage [22,23]. However, since changes in lamin expression determine nuclear stiffness [24,25], lamins also determine success at migration through confined spaces. An increased expression of lamins promotes nuclear stiffness and protects chromatin against mechanical damage, but also decreases migration through constrictions [11,13,22,26,27].

Chromatin contributes to nuclear rigidity through the ratio between euchromatin and heterochromatin, with heterochromatin being significantly stiffer than euchromatin [17,28]. The nuclear lamina and the euchromatin/heterochromatin ratio are both dynamic and respond to extracellular mechanical cues [18,19,29].

Mechanical cues are transmitted from the cellular environment to the nucleus through direct connections mediated by LINC (LInker of Nucleoskeleton and Cytoskeleton) complexes [30,31]. These complexes consist of a KASH-domain protein located in the outer nuclear membrane and a SUN-domain protein embedded in the inner nuclear membrane [31]. Humans express six KASH domain proteins (nesprins 1–4, KASH5, and LRMP). LINC complexes connect the nucleus to the cytoskeleton and convey forces across the nuclear membrane, altering nuclear stiffness and gene expression in response to environmental cues [18,32,33,34].

LINC complexes are important for cell motility on several levels. They are important for establishing the polarity of migrating cells, pushing the nucleus to the rear of the cell [35,36,37,38,39]. This polarization is consistent with a more efficient directional migration [40,41]. LINC complexes have also been implicated in contributing to cell rigidity. Cells with fewer LINC complexes are more malleable and can migrate more efficiently through constricted spaces, such as moving in and out of blood vessels [42].

Nesprin-4 is unique among the KASH domain proteins in its expression pattern, being restricted to highly polarized sensory hair cells in the inner ear [43] and to secretory epithelia, including the breast [44]. Nesprin-4 binds directly to kinesin-1 (Kif5A), the binding of which not only recruits kinesin to the nuclear envelope, but promotes activation of the kinesin [44,45]. Nesprin-4-dependent binding of kinesin at the nuclear envelope turns the nucleus into a kinesin cargo and drives cell polarization. In the inner ear hair cells, this nesprin-4-driven cell polarization is crucial for function and survival, and in the absence of nesprin-4, these cells fail to polarize, leading to cell death and an early-onset hearing loss in affected individuals [43].

The functional role of nesprin-4 in secretory epithelia has not been described. In this study we focus on breast tissue and show that nesprin-4 expression is elevated in breast cancer, though the levels of expression vary significantly between breast cancer subtypes. In less aggressive subtypes (luminal A, luminal B, and HER2-positive breast cancers), nesprin-4 is expressed at relatively high levels, while its expression is significantly reduced or completely lost in highly aggressive triple-negative breast cancer (TNBC). To understand the potential role for nesprin-4 in breast cancer, we used three breast cancer cell lines and modulated their nesprin-4 expression. We then assessed these cells in migration, invasion and intravasation assays. We show that nesprin-4 promotes migration and invasion in all cell lines. However, surprisingly, nesprin-4 blocks intravasation into an endothelial-based microvessel. We therefore propose that nesprin-4 has a dual role in breast cancer, promoting migration and invasion, but blocking intravasation.

## 2. Materials and Methods

### 2.1. Cell Line and Culture

Human breast cancer cell lines T47D, MCF7, and MDA-MB-231 (#HTB-133, #HTB-22, and #CRM-HTB-26, respectively, from ATCC) were cultured in Dulbecco’s Modified Eagle Medium (DMEM) supplemented with 10% (*v*/*v*) fetal bovine serum, 1% (*v*/*v*) L-glutamine, and 1% (*v*/*v*) penicillin and streptomycin. The cells were maintained at 37 °C in a humidified atmosphere containing 5% CO_2_.

### 2.2. Mycoplasma Testing

As part of our routine cell culture quality control, all cell lines were periodically screened for mycoplasma contamination using a PCR-based assay. Following the manufacturer’s instructions, we extracted DNA from 1 mL of cell culture supernatant using the QIAamp DNA Mini Kit (Qiagen, Hilden, Germany, Cat. #51306). For detection, we used a primer set targeting conserved regions of Mycoplasma DNA (Forward: 5′-GTGGGGAGCAAATAGGATTAGA-3′; Reverse: 5′-GGCATGTAGGTTTGAGGTC-3′). PCR reactions were prepared using AmpliTaq Gold™ Master Mix (Thermo Fisher Scientific, Foster city, CA, USA, Cat. #4398881), and thermal cycling was carried out under the following conditions: initial denaturation at 95 °C for 10 min; 35 cycles of 95 °C for 30 s, 53 °C for 30 s, and 72 °C for 30 s; followed by a final extension step at 72 °C for 7 min. Negative and positive controls were included in each run to validate the assay. No evidence of mycoplasma contamination was observed in any of the samples tested.

### 2.3. Plasmids and Transfections

Two GFP-labelled plasmids were used in this study. The PLV (shRNA)-EGFP plasmid (Vector Builder Inc., Chicago, IL, USA), specific against nesprin-4, was used to knock down endogenous nesprin-4 expression in T47D and MCF7. This shRNA targets a sequence near the 3’ end of the nesprin-4 (*SYNE4*) mRNA (CCTTATCCTCTTCCTCCTCTT). The PLV(Exp)-EGFP plasmid (Vector Builder Inc., Chicago, IL, USA) containing the *SYNE4* coding sequence was used to express the exogenous V5-tagged nesprin-4 in MDA-MB-231. The packaging cell line HEK-293 was transfected with both plasmids using lentiviral vectors through calcium phosphate transfection. After 48 h of transfection, lentivirus particles were harvested and used for the transduction of the cells. Breast cancer cell lines were transduced with lentivirus in the presence of polybrene (10 μg/mL) to enhance infection efficiency. Following transduction, cells were subjected to puromycin selection to enrich for successfully infected populations.

### 2.4. Quantitative Real-Time PCR

Total RNA was isolated from breast cancer cell lines using the RNeasy Plus Mini Kit (Qiagen, Hilden, Germany, Cat. #74106), following the manufacturer’s guidelines. Reverse transcription was performed using the High-Capacity cDNA Reverse Transcription Kit (Applied Biosystems, Thermo Fisher Scientific, Waltham, MA, USA, Cat. #4368813) to synthesize complementary DNA (cDNA) from the extracted RNA. The resulting cDNA was diluted 1:5 with nuclease-free water prior to amplification. Quantitative real-time PCR (RT-qPCR) was conducted using PowerUp™ SYBR™ Green Master Mix (Applied Biosystems Thermo Fisher Scientific, Waltham, MA, USA, Cat. #A25742) on a compatible real-time PCR system. The thermal cycling protocol consisted of an initial denaturation at 95 °C for 20 s, followed by 40 cycles of 95 °C for 1 s and 60 °C for 4 s. Gene expression levels of nesprin-4 were analyzed using the 2^−ΔΔCt^ method, with GAPDH serving as the endogenous reference gene to normalize expression data.

The primer sequences used for the PCR were as follows:

*SYNE4* (forward) 5′-TGGTCAACACCCTCTTCCTA-3′

*SYNE4* (reverse) 5′-TCTTCTGCTCAGCCTCTAGTA-3′

*GAPDH* (forward) 5′- CCCTTCATTGACCTCAACTACA -3′

*GAPDH* (reverse) 5′- CTGGAAGATGGTGATGGGATT -3′

### 2.5. Protein Detection by Immunoblotting

Protein lysates were separated on an 8% SDS-polyacrylamide gel and subsequently transferred onto a nitrocellulose membrane. To minimize nonspecific binding, the membrane was blocked for 1 h at room temperature using 3% BSA prepared in TBS containing 0.1% Tween-20 (TBST). Following the blocking step, the membrane was incubated at room temperature for 1.5 h with a rabbit polyclonal anti-nesprin-4 antibody (Proteintech, Rosemont, IL, USA, Cat. #26467-1-AP) diluted 1:300 in 3% BSA/TBST. The membrane was then incubated with HRP anti-rabbit secondary antibodies (1:10,000, Jackson, MS, USA, Cat. #115-035-003) for detection. Finally, the membrane was incubated with an ECL detection solution (Super-Signal West Dura, Thermo Fisher Scientific) and visualized using a ChemiDoc Imaging system (Bio-Rad, Hercules, CA, USA). GAPDH antibody (1:10,000, Cell Signaling, Danvers, MA, USA, Cat. #3683S) was used as a loading control to normalize the level of nesprin-4.

### 2.6. Immunofluorescence Microscopy

Cells cultured on sterile glass coverslips were fixed with 4% paraformaldehyde for 10 min at room temperature, followed by permeabilization with 0.1% Triton X-100 in PBS for 5 min. The permeabilized cells were blocked with 10% normal donkey serum (NDS) in 0.1% Triton X-100 in PBS for 1 h at RT. Primary antibody incubation was carried out for 1 h at room temperature. The primary antibodies used were as follows: rabbit anti-nesprin-4 antibody (1:500, Invitrogen, Carlsbad, CA, USA, Cat. #PA5-42661) to label the endogenous nesprin-4 in T47D-WT, T47D-N4-KD, MCF7 WT, and MCF7-N4-KD, and mouse anti-V5 tag antibody (1:250, Invitrogen, Thermo Fisher Scientific, Carlsbad, CA, USA, Cat. #46-0705) to label the exogenous nesprin-4 in nesprin-4-overexpressed MDA-MB-231. Mouse anti-alpha-tubulin antibody (1:250, ZooMAb® clone DM1A, Sigma-Aldrich, St. Louis, MO, USA, Cat. #ZMS1039) against tubulin alpha-1A chain of the microtubules and rabbit anti-pericentrin antibody (1:2000, Abcam, Cambridge, UK, Cat. #AB220784) to label centrosome; and mouse monoclonal antibody against human VE-Cadherin (1:30, Santa Cruz Biotechnology, Dallas, TX, USA, Cat. # SC-9989) was used to visualize cell–cell junctions. After primary antibody incubation, coverslips or chips were washed with PBS and incubated with the fluorescent-conjugated secondary antibodies. The secondary antibodies used were donkey anti-rabbit Alexa Fluor 647 (1:250, Thermo Fischer Scientific, Waltham, MA, USA, Cat. #A31573), donkey anti-mouse Alexa Fluor 568 (1:250, Thermo Fischer Scientific, Waltham, MA, USA, Cat. #A10037), and donkey anti-rabbit Alexa Fluor 488 (1:250, Thermo Fischer Scientific, Waltham, MA, USA, Cat. #A21206). Hoechst 33342 (Thermo Fisher Scientific, Waltham, MA, USA, Cat. #H3569) or DAPI (1:250, Thermo Scientific, Darmstadt, Germany, Cat. #62248) was used to stain the nuclei. For visualization of the actin filaments, Alexa Fluor 488 phalloidin stain (400x, Thermo Scientific, Waltham, MA, USA, Cat. #A12379) was mixed with the secondary antibody, and cell bodies were visualized using CellMask™ Deep Red stain (Thermo Fisher Scientific, Waltham, MA, USA, Cat. #H32721), which stains the entire cell.. Finally, coverslips were mounted using ProLong™ Diamond Antifade mounting media (Thermo Fisher Scientific, Eugene, OR, USA, Cat. #P36961), and fluorescence images were acquired using a Nikon A1 confocal laser scanning microscope (Nikon, Minato-ku, Tokyo, Japan).

### 2.7. Migration Assays

Cell migration was evaluated using two distinct approaches: the scratch wound healing assay and the Oris™ cell migration assay. In the scratch wound healing assay, T47D, MCF7, and MDA-MB-231 cells were seeded in a 6-well plate. The plate was incubated in a confluent monolayer. The scratch wounds were made in the monolayer using a 1 mL syringe tip, washed twice with DMEM to remove debris, and then incubated in a culture medium supplemented with 10% FBS. Images of the scratch area were taken at 0 h, 24 h, and 48 h using the EVOS microscope. In the Oris^TM^ cell migration assay, the cells were seeded in a 96-well plate at 8×10^5^ cells/well. The plate was populated with silicone stoppers to create regular round clear zones. As soon as the confluent monolayer was formed, all stoppers were removed, and the wells were washed twice with sterile PBS, the Oris™ assay plates were placed in a 37 °C incubator with 5% CO_2_ and high humidity to promote cell migration. Images at 0 h, 24 h, and 48 h were acquired using the Operetta CLS™ high-content analysis system. For both assays, cell movement was assessed by analyzing the images captured before and after migration using Fiji (ImageJ, version 1.53t, Java 1.8.0_322 (64-bit), National Institutes of Health, USA; https://imagej.nih.gov/ij/, accessed on 21 August 2021) to quantify the area covered over time. Cell migration was quantified by calculating the percentage of wound closure using the following formula: [(pre-migration area) − (migration area)/(pre-migration area) × 100].

### 2.8. Transwell Invasion Assay

Cell invasion was carried out using transwell invasion chambers (PET-membrane, 8.0 µm, cell Grade™ plus, BRAND, Wertheim, Germany) that were coated with two different matrices, Geltrex^TM^ LDEV-Free matrix (Gibco™, Thermo Fisher Scientific, Waltham, MA, USA, Cat. #A1413302; diluted 1:4 with cold serum-free DMEM) and Corning^®^ Matrigel^®^ Growth Factor Reduced (GFR) Basement Membrane Matrix, LDEV-free (Corning Inc., Corning, NY, USA, Cat. #356230; diluted 1:1 with cold serum-free DMEM). The third matrix used is the ECMatrix™ (Sigma-Aldrich, Merck KGaA, Darmstadt, Germany, Cat. #ECM550; used as provided in the CHEMICON Cell Invasion Assay Kit) as a 24-well tissue culture plate with 12 cell culture inserts (8 μm pore size polycarbonate membrane). The cells were seeded in the upper chamber at a 5×10^5^ cells/mL density in 100 µL serum-free medium. The lower chamber was filled with 650 µL DMEM containing 10% FBS and 50 ng/mL epidermal growth factor (EGF) as a chemoattractant. The chambers for MCF7 and T47D were incubated for 3–4 days, and for 24 h for MDA-MB-231, at 37 °C to allow the cells to migrate. After incubation, the non-invading cells on the top surface of the matrix membrane were removed carefully with sterile cotton swabs. To assess cell invasion, cells that had traversed to the underside of the insert membrane were stained with 0.2% crystal violet dissolved in methanol for 30 min. The bound crystal violet was solubilized using 33% acetic acid for 15 min. The resulting solution was transferred to a clear 96-well plate, and the optical density was measured at 590 nm using a TECAN microplate reader.

### 2.9. EdU Cell Proliferation Assay

Cell proliferation was evaluated using the Click-iT^®^ EdU Imaging Kit with Alexa Fluor™ 594 dye (Thermo Fisher Scientific, Waltham, MA, USA, Cat. #C10339). Cells were seeded into 96-well plates and incubated overnight at 37 °C. The next day, an equal volume of 20 μM EdU solution was added to the wells, resulting in a final EdU concentration of 10 μM, and the cells were further incubated for 3 h in a humidified incubator at 37 °C with 5% CO_2_. Following incubation, cells were fixed with 4% paraformaldehyde in PBS for 15 min at room temperature, then permeabilized with 0.5% Triton^®^ X-100 in PBS for 20 min. After a brief wash with 3% BSA in PBS, the Click-iT^®^ reaction cocktail was applied for 30 min at room temperature to detect EdU incorporation. To visualize cell nuclei, cells were stained with Hoechst^®^ 33342 (1:2000 dilution) for 30 min. Imaging was performed using the Operetta CLS™ high-content analysis system, and the percentage of EdU-positive cells was quantified as a measure of proliferation.

### 2.10. Organ-on-Chip Assay

An Organo Plate^®^ 3-lane platform (Mimetas, Leiden, Netherland, Cat. #6405-400-B) was utilized to model and quantify the invasive and intravasative behavior of breast cancer cells under dynamic flow conditions. To initiate the setup, 2.2 μL of 5 mg/mL collagen I was introduced into the central ECM channel of the chip. The plate was then placed in a humidified incubator at 37 °C with 5% CO_2_ for 15 min to allow the collagen to polymerize. Following matrix solidification, 40 μL of 10 μg/mL fibronectin (prepared in PBS) was added to the inlets of the two adjacent perfusion lanes to enhance cell adhesion.

The following day, endothelial cells were suspended at a density of 10,000 cells/μL in Endothelial Cell Growth Medium-2 (EGM-2) and seeded into one of the lateral channels designated for vascular formation. The plate was gently tilted in the incubator for 2–3 h to promote uniform cell attachment. After this period, 50 μL of EGM-2 was added to both ends of the vascular channel. The plate was then transferred to the Mimetas rocker platform, where a bi-directional pulsatile flow was applied to simulate physiological shear stress (2.5 dyne/cm^2^) at 8-min intervals and at a 14° angle. The culture medium was replenished every 48 h. Within 4 days, a confluent endothelial tube mimicking a microvessel had formed.

For tumor introduction, a cell suspension containing GFP-tagged breast cancer cells and adult dermal fibroblasts (both at 4 × 10^6^ cells/mL) was prepared in DMEM. Three microliters of this mixture were seeded into the opposite perfusion lane (tumor channel), and the plate was again incubated for 2–3 h to facilitate cell attachment. Following this, 50 μL of DMEM was added to both the inlet and outlet of the tumor lane. Throughout the experiment, endothelial cells in the vascular channel were maintained in EGM-2, while tumor cells and fibroblasts in the tumor lane were cultured in DMEM. The chip remained on the rocking platform to maintain dynamic conditions. After a designated incubation period, invasion and intravasation events were assessed using a high-content imaging system as previously described [46].

### 2.11. Data Acquisition and Statistical Analysis

Nesprin-4 expression data were derived from the Oncomine gene expression array database (OncoDB) (https://oncodb.org, accessed on 25 February 2024.) The survival analysis and the Kaplan–Meier plots were performed using the Kaplan–Meier plotter (https://kmplot.com/analysis/index.php?p=service&cancer=breast, accessed on 18 March 2024). Nesprin-4 expressions in normal breast tissues and tumor tissues were derived from the Gene Expression Database (GENT2) (http://gent2.appex.kr/gent2/, accessed on 25 February 2024). The Human Protein Atlas (HPA) was utilized to assess the relative expression of nesprin-4 in normal breast tissues and breast cancer tissues (https://www.proteinatlas.org, 12 September 2024). Rose plots were created using Rose Diagram Maker (https://geographyfieldwork.com/RoseDiagramCreator.html, accessed on 10 October 2024). Statistical analyses were performed using GraphPad Prism version 10 (GraphPad Software). All quantitative results are expressed as the mean ± standard deviation (SD). To assess statistical significance between two groups, an unpaired Student’s t-test was applied, with *P* < 0.05 considered statistically significant.

## 3. Results

### 3.1. Nesprin-4 Expression Is Elevated in Breast Cancer

**Figure 1:**
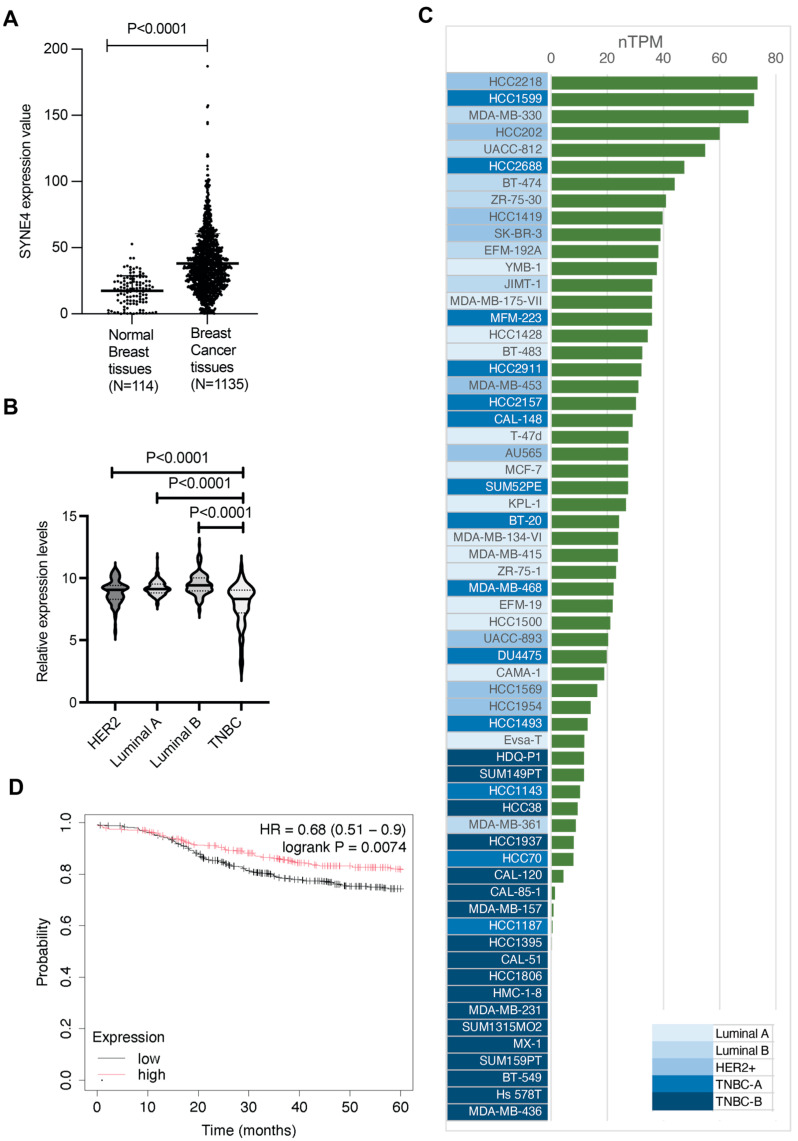
Differential expression and clinical relevance of nesprin-4 in breast cancer. (**A**) Normalized transcript levels of nesprin-4 were compared between breast cancer tissues (*n* = 1135) and normal breast tissues (*n* = 114). The data, retrieved from the OncoDB database, show a significant increase in nesprin-4 expression in tumor samples (*P* < 0.0001), presented as normalized transcripts per million (nTPM). (**B**) A violin plot illustrates the variability in nesprin-4 expression among breast cancer molecular subtypes: Luminal A (*n* = 188), Luminal B (*n* = 137), HER2-positive (*n* = 137), and TNBC (*n* = 252). Statistically significant differences were observed between TNBC and the other subtypes (Luminal A vs. TNBC, *P* < 0.0001; Luminal B vs. TNBC, *P* < 0.0001; HER2 vs. TNBC, *P* < 0.0001). (**C**) A bar chart displays nesprin-4 expression profiles in 62 human breast cancer cell lines, categorized by molecular subtype: Luminal A, Luminal B, HER2-positive, and TNBC. Expression levels (nTPM) are shown in green bars and ranked in descending order. Background shading in varying intensities of blue indicates increasing subtype aggressiveness, with dark blue marking the most aggressive group (TNBC-B). (**D**) Kaplan–Meier analysis of distant metastasis-free survival (DMFS) over 5 years in 2765 patients revealed a significant survival advantage for patients with high nesprin-4 expression compared to those with low expression (HR = 0.68; 95% CI: 0.51–0.9; log-rank *P* = 0.0074).

We have observed that different breast cancer cell lines (MCF-7, T47D and MDA-MB-231) had different levels of nesprin-4 expression (unpublished): MCF-7 and T47D express nesprin-4 endogenously, while MDA-MB-231 cells do not. To understand whether these differences might be important, we examined the expression databases of patient samples. Using the Oncomine gene expression database (https://oncodb.org, accessed on 25 February 2024), we found that *SYNE4* (nesprin-4) mRNA levels in breast cancer tissues are significantly upregulated in invasive carcinoma compared to normal breast tissue (*P* < 0.0001) (Figure 1A).

Breast cancer is categorized according to standard molecular criteria: Luminal A, Luminal B, HER2-enriched, and Triple-Negative Breast Cancer (TNBC), as defined by ER, PR, and HER2 status [47,48,49,50]. These subtypes exhibit different levels of aggressiveness, with Luminal A being the least aggressive, followed by Luminal B and HER2+ [49,51]. TNBC is recognized as the most aggressive breast cancer subtype and can be further categorized into subtypes A and B, with subtype B being the most aggressive [51,52]. We wanted to determine if the nesprin-4 expression levels correlated with any of these subtypes. Using data from the Gene Expression Database of Normal and Tumor Tissues (GENT2) (http://gent2.appex.kr/gent2/, accessed on 25 February 2024) we found that nesprin-4 expression is significantly reduced in TNBCs compared to each of the other subtypes (*P* < 0.0001) (Figure 1B).

Breast cancer cell lines have been used to model the disease for decades, and many have well-characterized expression profiles. Using the Human Protein Atlas data, we queried the nesprin-4 expression in a panel of 62 breast cancer cell lines from different subtypes (http://www.proteinatlas.org, accessed on 12 September 2024). We arranged the cell lines according to expression levels and then classified the cell lines into the different subtypes, as previously published [51]. The data show a trend where the least aggressive cells have the highest nesprin-4 expression (luminal (A and B) and HER2+ subtypes) and the more aggressive TNBC have either low or no nesprin-4 expression. When we further subdivided TNBC, we found that the most aggressive TNBC subtype B cell lines all clustered at the bottom of the graph, with most of these (16/18) showing a normalized expression level (nTPM) of less than 10 (Figure 1C). These cell line data mirror the nesprin-4 expression in clinical samples (Figure 1B). We also examined the expression of Nesprins-1, -2, and -3 in the same cohort of breast cancer cell lines but did not observe a similar trend (Figure S1).

Finally, we wanted to determine whether the levels of nesprin-4 expression affect the clinical outcome for breast cancer patients. We used the Kaplan–Meier Plotter (https://kmplot.com/analysis/index.php?p=service&cancer=breast, accessed on 18 March 2024) and found that patients with high nesprin-4 expression exhibited slightly poorer overall survival (OS) compared to those with low expression (HR = 1.16, 95% CI: 0.89–1.52) (Figure S2), though this was not statistically significant. However, we found that high nesprin-4 levels were associated with a better distant metastasis-free survival (DMFS) at five years post-diagnosis (*P* = 0.0074) (Figure 1D). This advantage is temporary, and at the 10-year time point, the protective effect of elevated nesprin-4 is lost (Figure S3).

### 3.2. Nesprin-4 Expression Levels in Breast Cancer Cell Lines

**Figure 2:**
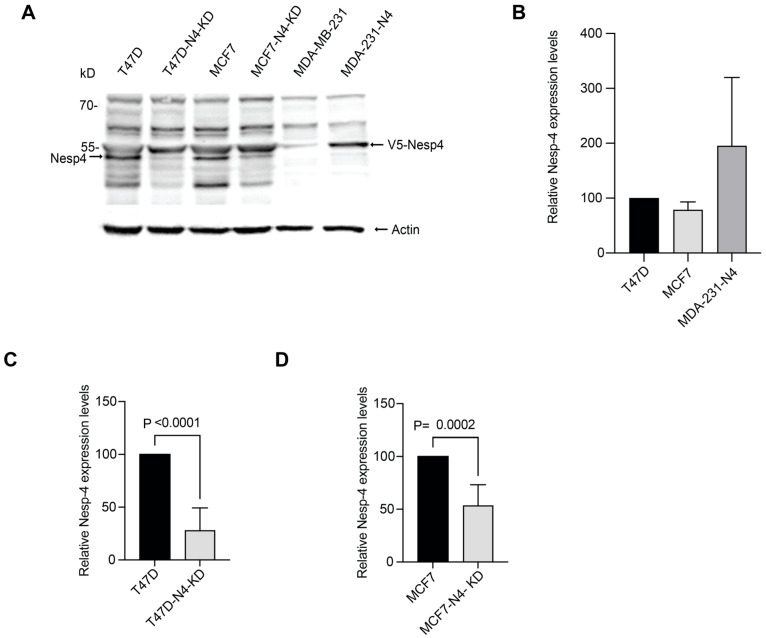
Differential nesprin-4 protein levels across breast cancer cell lines. (**A**) Immunoblot of breast cancer cell lines using anti-nesprin-4 antibody. Nesprin-4 was knocked down in T47D (T47D-N4-KD) and MCF7 (MCF7-N4-KD) cell lines and overexpressed in MDA-MB-231 (MDA-231-N4). The molecular weight of the endogenous nesprin-4 in wild-type T47D and MCF7 is ~42 kDa and appears in the blot at ~50 kDa (as indicated by the arrow) due to its high proline content, as previously reported [45]. In MDA-MB-231, the exogenous V5 tagged nesprin-4 appears at ~55 kDa. We used actin as a loading control (lower blot). Immunoblot bands were digitally quantified using ImageJ software. (**B**) Shows the relative protein expression levels of nesprin-4 across three different cell lines: T47D, MCF7, and MDA-231-N4, with expression normalized to T47D. (**C**) Comparison of wild-type and nesprin-4 knockdown in T47D cells. Expression is normalized against endogenous nesprin-4. (**D**) Comparison of wild-type and nesprin-4 knockdown in MCF7 cells. Expression is normalized against endogenous nesprin-4. Quantifications in (**B**–**D**) were performed on four separate immunoblots, representing four biological replicates.

To investigate the functional significance of nesprin-4 in breast cancer, we used three well-established and commonly used breast cancer cell lines: T47D and MCF7, which are classified as Luminal A subtypes and MDA-MB-231, a highly invasive cell line that models triple-negative breast cancer (TNBC) subtype B [51]. An immunoblot analysis for nesprin-4 expression in these cells showed nesprin-4 expression in T47D and MCF7 cell lysates, but no detectable nesprin-4 expression in MDA-MB-231 (Figure 2A). We used nesprin-4 shRNA to knock down nesprin-4 expression in T47D (T47D-N4-KD) and MCF7 (MCF7-N4-KD) cells. We used a V5-tagged construct to overexpress nesprin-4 in MDA-MB-231 (MDA-231-N4). The relative expression levels of nesprin-4 were confirmed by immunoblotting whole cell lysates (Figure 2A), and the blots were digitally quantified. The nesprin-4 levels in the MDA-231-N4 were slightly higher (less than 2X) than the endogenous expression of nesprin-4 in T47D and MCF-7 cells (Figure 2B). Nesprin-4 knockdown was more efficient in T47D cells (Figure 2C) compared to MCF7 cells (Figure 2D), though both lines exhibited a notable reduction in expression relative to their respective wild-type controls. The expression data for these six cell lines were also confirmed through an RT-qPCR analysis (Figure S4) and immunofluorescence imaging (Figure S5), which support the immunoblot data.

### 3.3. Nesprin-4 Promotes Two-Dimensional (2D) Cell Migration

**Figure 3:**
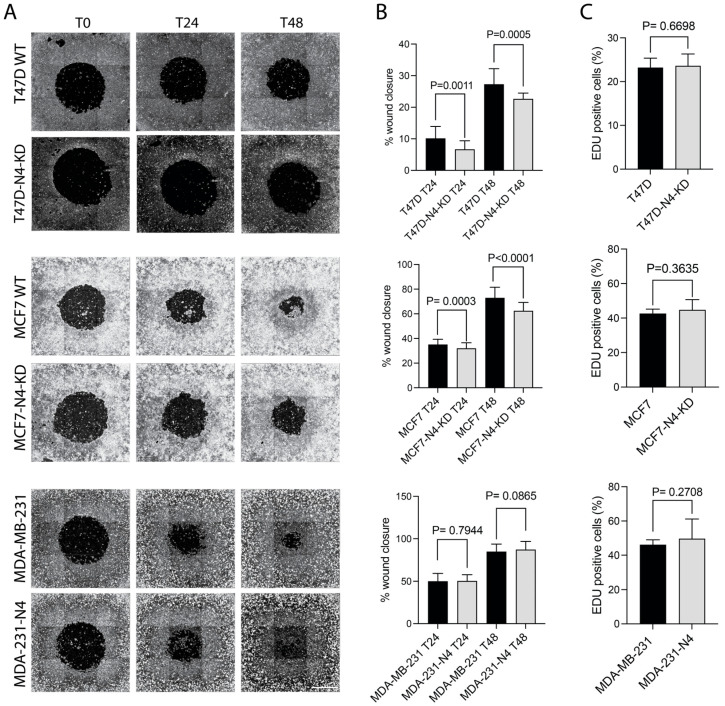
Effect of nesprin-4 on 2D cell migration. (**A**) An Oris^TM^ cell migration assay was performed by making regular zones for cell migration using silicon stoppers. (**B**) The analysis revealed a significant reduction in cell migration in the nesprin-4 knockdown of T47D cells compared to wild-type controls at both 24 h (*P* = 0.0011) and 48 h (*P* = 0.0005). A similar trend was observed in MCF7 cells, where the knockdown of nesprin-4 also led to a significant decrease in migration at 24 h (*P* = 0.0003) and 48 h (*P* < 0.0001). By contrast, nesprin-4 expression did not impact the migration of MDA-MB-231 cells at either 24 h (*P* = 0.7944) or 48 h (*P* = 0.0865). Migration was quantified as the percentage of wound closure, calculated using the formula: (pre-migration area) − (migration area)/(pre-migration area) × 100. (**C**) An EdU (5′-ethynyl-2′-deoxyuridine) incorporation assay was performed on all cell lines.

Nesprin-4 has been shown to bind kinesin-1 and to affect cell polarization in non-migrating cells by affecting nuclear and centrosomal positioning within the cell [43,44]. In fact, in the absence of nesprin-4, sensory hair cells in the inner ear are unable to develop a fully polarized phenotype, leading to hearing loss [43]. Polarization is also an important step in cell migration. To investigate whether nesprin-4 affects breast cancer cell migration, we first examined 2D cell migration. We used the Oris^TM^ cell migration assay to create regular circular wounds in a 96-well plate. Imaging was performed using the Operetta^®^ High Content Imaging System at three time points: immediately after gap creation (T0), and again at 24 and 48 h (T24 and T48) (Figure 3A). The wound area was measured at each time point, and the wound closure rate was subsequently calculated (Figure 3B). We found that nesprin-4 expression significantly enhanced wound closure in both MCF7 and T47D cells at 24-h and 48-h following wounding (Figure 3B, top two graphs). In MDA-MB-231 cells, nesprin-4 expression had no observable effect on 2D migration (Figure 3B, bottom graph). To exclude the role of cell proliferation in the observed differences in wound closure, we performed an EdU (5′-ethynyl-2′-deoxyuridine) incorporation assay. We found that nesprin-4 did not affect cell proliferation (Figure 3C).

Therefore, we conclude that the observed changes in wound closure are a result of changes in cell motility. We obtained similar results using the linear scratch wound assay (Figure S6). These findings suggest that nesprin-4 positively contributes to the mobility of Luminal A-type breast cancer cell lines. By contrast, TNBC MDA-MB-231 cells were not affected by the presence of nesprin-4 in 2D migration.

### 3.4. Nesprin-4 Promotes Three-Dimensional (3D) Invasion

**Figure 4:**
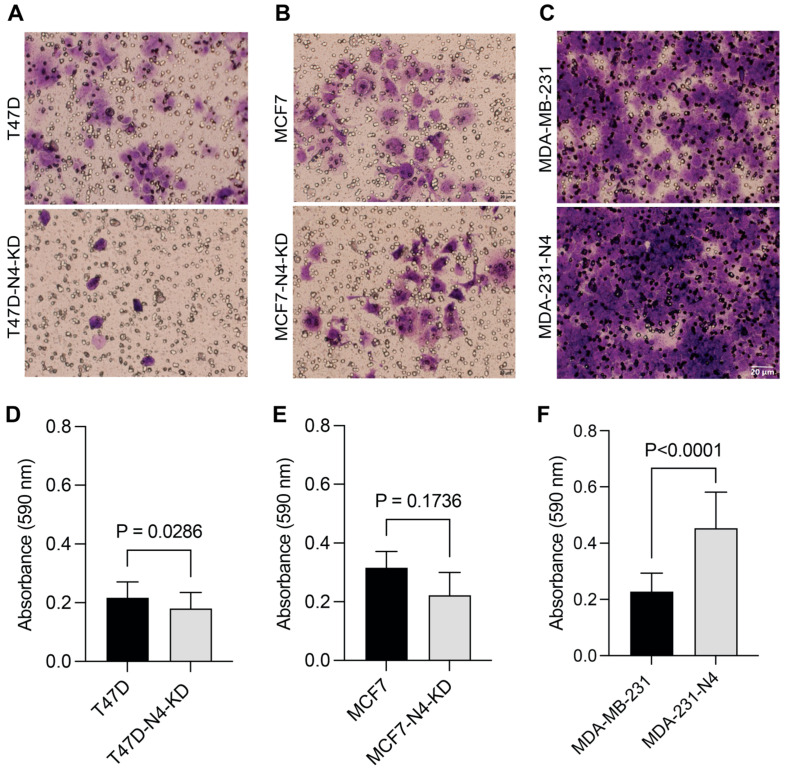
Nesprin-4 increases the ECM invasion of breast cancer cells. T47D, MCF7, MDA-MB-231, and their corresponding nesprin-4-modified derivatives were seeded onto extracellular matrix (ECM) gels in serum-free media. The lower chamber contained DMEM supplemented with 10% fetal bovine serum (FBS) and 50 ng/mL EGF to serve as a chemoattractant. After incubation, cells that had migrated through the ECM and adhered to the underside of the insert were stained using 0.2% crystal violet and subsequently imaged. Representative images of the invaded cells are presented in (**A**–**C**). Quantification of the invasion is shown in (**D**–**F**). Bound dye was solubilized with acetic acid, and the optical density was measured to quantify invasion. The bar graphs represent the mean values from at least four independent biological replicates.

We then wanted to determine whether nesprin-4 affects 3D migration and invasion. We plated cells on an extracellular matrix (ECM) in transwell inserts with an 8 μm pore size. Cells that successfully invaded through the ECM towards a growth factor gradient were stained with crystal violet, and the number of invaded cells was quantified. Representative images of each cell line are shown in (Figure 4A). The crystal violet dye was solubilized from cells at the bottom of the membrane, and the absorbance was measured (Figure 4B). We found that nesprin-4 significantly promotes 3D invasion in T47D (*P* = 0.0286) and MDA-MB-231 cells (*P* < 0.0001). In MCF7 cells, nesprin-4 trended (*P* = 0.1736) towards increasing the 3D invasive capacity of these cells. We found similar results for a 3D migration assay using a Boyden chamber without an ECM (Figure S7). Taken together our results indicate that nesprin-4 promotes 3D migration and invasion in T47D, MCF7, and MDA-MB-231 breast cancer cell lines.

### 3.5. Nesprin-4 Affects Nuclear Positioning During Breast Cancer Cell Migration

Having shown that nesprin-4 can impact migration and invasion of breast cancer cells, we wanted to understand the mechanism behind this increase in motility. Efficient cell migration depends on a series of cellular mechanisms that promote directional movement [53]. When cells become motile, the nucleus shifts toward the rear of the cell, creating a clear distinction between the leading edge and the trailing end [54]. Nesprin-4 is known to bind kinesin-1 and, through this interaction, has been shown to promote nuclear and centrosomal polarization in cells and tissues [43,44], suggesting that nesprin-4 may affect migration through polarization.

**Figure 5:**
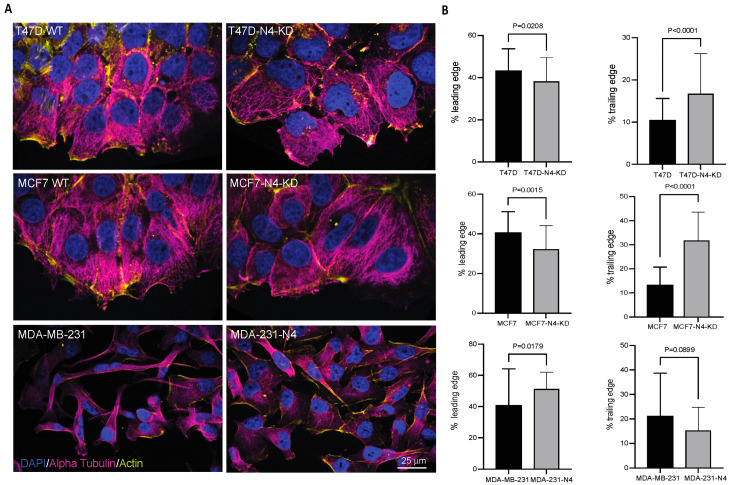
Nesprin-4 affects cell polarization in directional migration. (**A**) Confocal images of the three breast cancer cell lines with modulated nesprin-4 expression. Confluent cell monolayers were scratched to create a wound, and cells were allowed to migrate into this space. The wound edge is positioned at the bottom of each panel. Cells were stained for alpha-tubulin (magenta), nuclei with DAPI (blue), and actin filaments with phalloidin (yellow). Scale bar represents 25 μm. (**B**) The lengths of the leading and trailing edges of migrating cells were quantified using ImageJ software, and data were plotted using Prism. The leading-edge distance was measured from the front of the cell to the nucleus, while the trailing edge was measured from the nucleus to the cell rear. Percentages were calculated by dividing each respective edge length by the total cell length and multiplying by 100 (i.e., leading edge % = (leading edge length/total cell length) × 100; trailing edge % = (trailing edge length/total cell length) × 100). Values are expressed as mean ± SEM with sample sizes: T47D (*n* = 58), T47D knockdown (*n* = 51), MCF7 (*n* = 40), MCF7 knockdown (*n* = 45), MDA-MB-231 (*n* = 32), and MDA-MB-231 overexpressing nesprin-4 (*n* = 36).

To investigate whether nesprin-4 contributes to polarization in migrating breast cancer cells, we scratched a confluent monolayer of cells and allowed the cells time to establish directional migration into the wound. Cells were then fixed and stained for nuclei, microtubules, and actin (Figure 5A). For the analysis, we selected cells at the edge of the wound and measured the distances in front of the nucleus (leading edge) and behind the nucleus (trailing edge). All cells expressing nesprin-4 had a significantly longer leading edge than cells with reduced/no nesprin-4 expression (Figure 5B, left graphs). We also saw a significantly shorter trailing edge in MCF7 and T47D cells compared to their nesprin-4 KD counterparts (Figure 5B). The MDA-231-N4 cells showed a trend towards a shorter trailing edge in cells compared to the parental MDA-MB-231 cells (*P* = 0.0899) (Figure 5B, right graphs). These results indicate that, in breast cancer cell lines, nesprin-4 promotes cell polarization during 2D directional migration.

### 3.6. Nesprin-4 Expression Affects Centrosome Localization in Breast Cancer Cells

**Figure 6:**
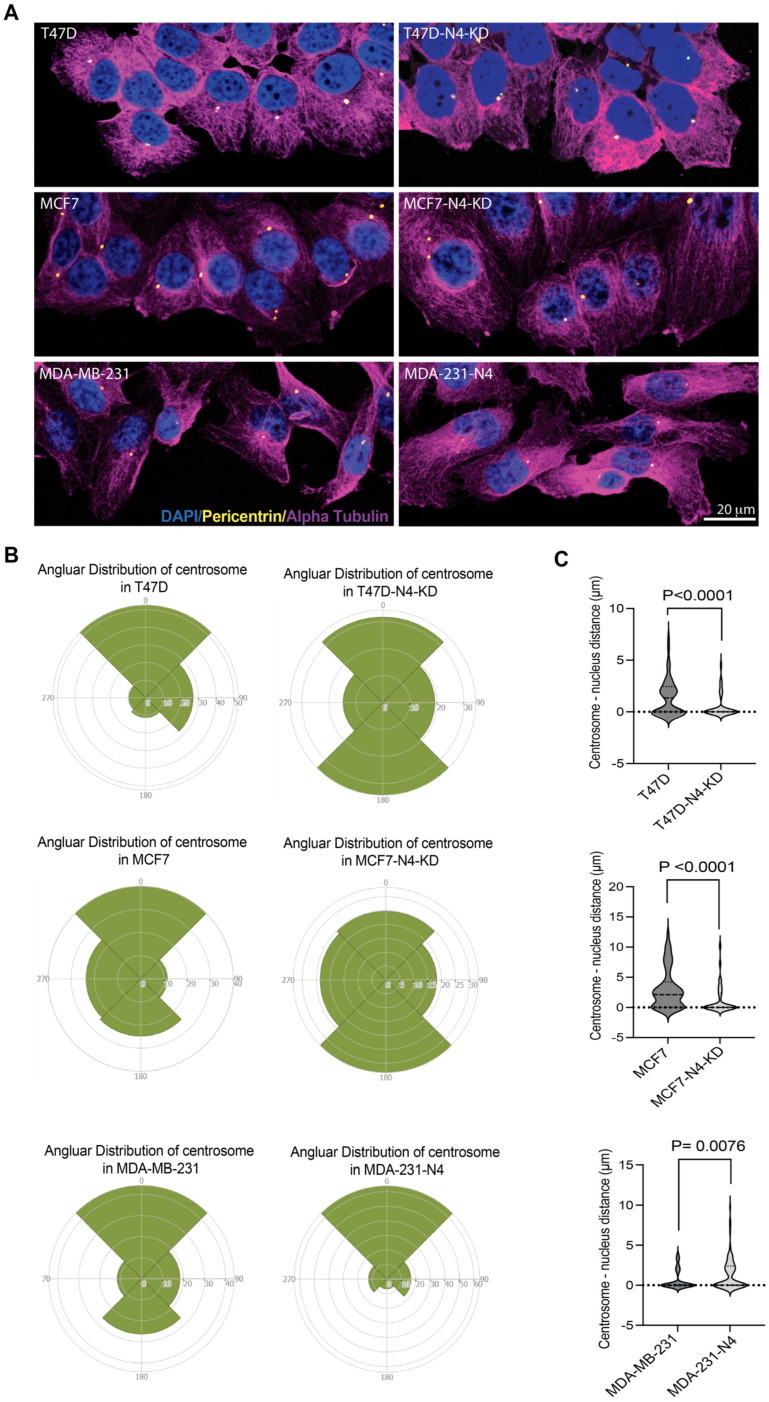
Nesprin-4 expression affects localization of the centrosome in migrating cells. (**A**) Immunofluorescence images of breast cancer cells migrating into a wound (at the bottom of each image) and immunostained for centrosomes (anti-Pericentrin antibody, Yellow), microtubules (Anti-alpha-tubulin, Magenta), and the nuclei (DAPI, Blue), scale bar 20 μm. (**B**) The rose plot demonstrates the angular distribution of centrosomes in breast cancer cells relative to the nucleus and migration direction (0°), each rose plot is divided into four quadrants: 0° (direction of migration, leading edge), 90°, 180°, and 270° (trailing edge). The percentage of centrosomes located within each quadrant is represented by the area covered in the corresponding section of the rose plot. (**C**) Violin plots demonstrate the centrosome–nucleus distance. The distance between the centrosome and the nearest point on the nuclear periphery was measured using Image J and graphed as violin plots.

In addition to the rearward positioning of the nucleus, most migrating cells position the centrosome ahead of the nucleus towards the leading edge [41]. Previous work has shown that nesprin-4 plays a role in centrosomal positioning, particularly affecting the distance between the centrosome and the nucleus [44]. To determine whether nesprin-4 affects centrosome localization in migrating breast cancer cells, we scratched a confluent monolayer of cells and fixed cells once directional migration was well established. Cells were stained with a centrosome marker (anti-pericentrin), DAPI, and tubulin (anti-alpha-tubulin) (Figure 6A). Based on cell morphology, we could assign directionality to each cell (0° = direction of migration). We then divided each cell into four quadrants centered on the nucleus: 315° to 45° (45° on either side of 0°), 45° to 135°, 135° to 225°, and 225° to 315° quadrants (Figure 6B). The centrosome location in each cell was assigned into one of these four quadrants. The percentage of centrosomes in each quadrant was plotted on a rose plot using Rose Diagram Maker to show the angular distribution of the centrosome relative to the direction of migration (Figure 6B). In T47D cells, the highest percentage of centrosomes (52.5%) was concentrated in the leading-edge quadrant, while in T47D-N4-KD cells, the highest percentage of centrosomes (68.2%) was dispersed in the non-leading edges. MCF7 cells also showed a centrosome orientation toward the leading-edge quadrant (36.4%), and upon nesprin-4 knockdown, the centrosome distribution became more dispersed, reducing the percentage of cells with leading-edge centrosomes to 24.7%. In MDA-MB-231 cells, the centrosomes were distributed across the quadrants, with (44.2%) of them orienting to the leading edge. In response to nesprin-4 expression, centrosome orientation in the leading edge increased to 63.9%, a value that is similar to that of T47D and MCF7 cells. These results indicate that breast cancer cells expressing nesprin-4 (T47D, MCF7, and MDA-231-N4) have more centrosomes focused toward the leading edge, while in nesprin-4-knockdown or nesprin-4-non-expressing cells, the centrosome localization is more dispersed. We also analyzed these results by dividing cells into only two areas, leading edge and trailing edge. We observed the same trend in that nesprin-4 promotes leading edge localization of centrosomes (Figure S8).

Since nesprin-4 expression affects the distance between the centrosome and the nucleus in HeLa cells [44], we wanted to determine if we could observe a similar phenomenon in breast cancer cell lines. We measured the distance between the centrosome and the nucleus using ImageJ software. The results showed a statistically significant increase in centrosome–nucleus distance in nesprin-4-expressing cells compared to cells with reduced or no nesprin-4 expression (Figure 6C). The average centrosome–nucleus distance in wild-type T47D cells was ~1.5 μm and decreased to 0.57 μm in T47D-N4-KD cells (*P* < 0.0001). The average centrosome–nucleus distance in wild-type MCF7 was 3.1 μm and decreased to 1.3 μm upon knockdown of nesprin-4 (*P* < 0.0001). Overexpression of nesprin-4 in MDA-MB-231 increased the average centrosome–nucleus distance from 0.77 μm to 1.4 μm (*P* = 0.0076). These findings indicate that, in breast cancer cells, nesprin-4 promotes centrosome orientation and positioning. Thus, the nesprin-4-dependent relocalization of the nucleus and centrosome may support directional migration in breast cancer cells.

### 3.7. Nesprin-4 Promotes Invasion but Blocks Intravasation in a Metastasis-on-Chip Model

**Figure 7:**
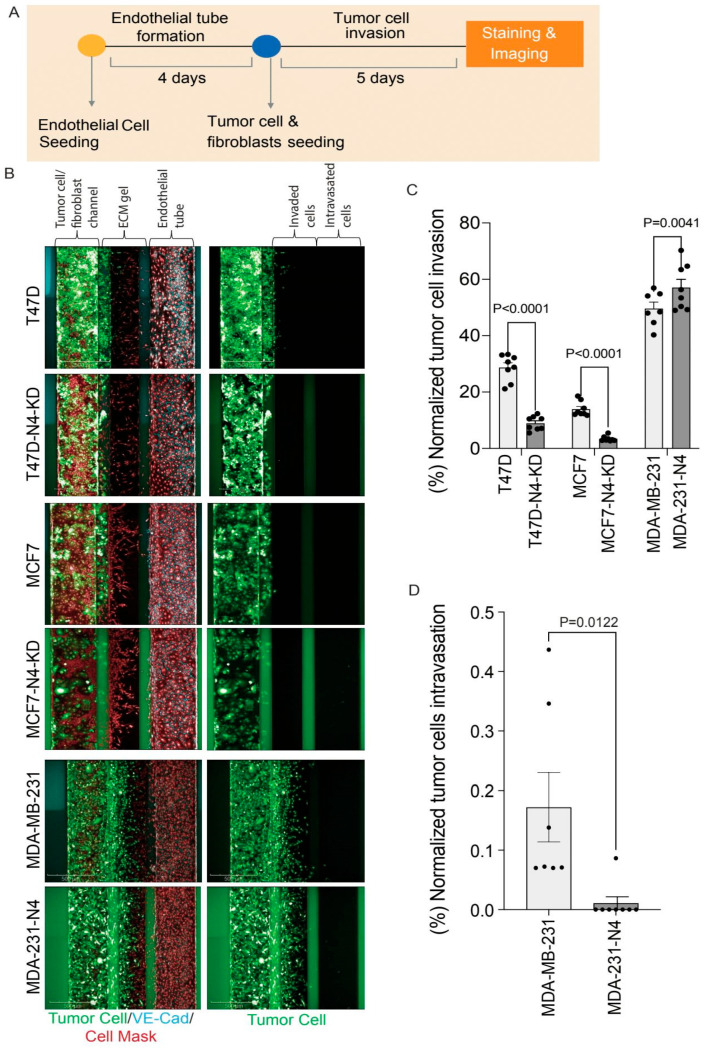
Nesprin-4 promotes breast cancer cell invasion but blocks intravasation in an organ-on-chip model. (**A**) Schematic representing the workflow for the organ-on-chip assay. Endothelial cells were grown for four days to allow them to organize into tube-like structures. Tumor cells and fibroblasts were then seeded and monitored over a period of 5 days. (**B**) Fluorescent images were taken on day 5 after tumor cell seeding. The three areas of each channel are shown at the top left, with green fluorescence representing tumor cells (labelled with a GFP marker), blue endothelial cell junctions (stained with a VE-Cadherin marker), and red showing fibroblasts and endothelial cells (cell mask). The right channels show tumor cells (GFP) only. The images were taken using the Operetta CLS high-content imaging platform (10× objective lens). (**C**) Shows the quantification of tumor cell invasion as a percentage of normalized invaded tumor cells to total tumor cell number in the channel. There is a significant decrease in cell invasion in nesprin-4 knockdown T47D and MCF7 compared to the wild-type T47D (*P* < 0.0001) and MCF7 (*P* < 0.0001) cells. MDA-MB-231 cells show a significant increase in cell invasion when nesprin-4 is expressed (*P* = 0.0041). (**D**) Shows the percentage of normalized tumor cell intravasation to total tumor cell number in the channel. In MDA-MB-231, expression of nesprin-4 reduces the intravasation through the endothelial tube barrier (*P* = 0.0122). Each graph in **C** and **D** represents the average of eight biological replicates.

Cancer metastasis is a multistep process that involves invasion of the surrounding tissues, intravasation into blood vessels, extravasation at distal sites, and the formation of secondary tumors at these sites [6,55,56]. A critical step in metastasis is the ability of tumor cells to penetrate the endothelial barrier and to gain access to the blood vessels. This step is a complex event that is mediated by the interactions between cancer cells, stromal fibroblasts, and endothelial cells [57]. Organ-on-chip models have advanced to mimic the microenvironment and physiological conditions of human tissues and to allow for a controlled and reproducible study of metastatic processes and tumor progression [46,58]. The organ-on-chip model used in this study [46] simulates a tumor microenvironment with an adjacent blood microvessel with flow. In this model, fibroblasts are co-cultured with the GFP-labelled breast cancer cell lines (T47D, T47D-N4-KD, MCF7, MCF7-N4-KD, MDA-MB-231, and MDA-231-N4) in a 1:1 ratio, in a channel separated from the endothelial tubes by an ECM (Figure 7A). This system has the great advantage that components of the metastatic cascade (migration, invasion, and intravasation) can be distinguished and quantitated separately.

To reach the endothelial tube, cells must first pass through the collagen gel that mimics the extracellular matrix (ECM) of tumors. Fluorescence imaging was used to monitor and quantify the invasion and intravasation of breast cancer cells, with and without nesprin-4 expression (Figure 7B). Nesprin-4 knockdown (N4-KD) significantly reduced tumor cell invasion in T47D and MCF7 cells (Figure 7C). In addition, ectopically expressing nesprin-4 in the already highly invasive MDA-MB-231 cells further increases invasion (Figure 7C). These observations align with the data presented in Figure 4B, where nesprin-4 was shown to enhance 3D invasion in both luminal and triple-negative breast cancer cell lines.

Next, using the same organ-on-chip assay, we investigated the tumor cell intravasation by monitoring the number of cells that successfully traverse the endothelial barrier. Neither T47D, MCF7, nor their derivative N4-KD cells invaded the ECM at a rate that was fast enough to reach the endothelial tube during the duration of this assay. We could not extend the assay period for longer, as integrity of the endothelial layer breaks down over time. Therefore, we could not measure intravasation for these cells. By contrast, MDA-MB-231 and MDA-231-N4 cells reached the microvessel border. Interestingly, the expression of nesprin-4 in MDA-MB-231 cells significantly decreased the ability of these cells to intravasate into the endothelial microvessels despite their increased invasiveness (*P* = 0.0122) (Figure 7D).

## 4. Discussion

In this study we report that changes in nesprin-4 expression are associated with breast cancer progression. In most breast cancers, Neprin-4 expression is elevated compared to normal breast tissue. However, in triple-negative breast cancers (TNBC), nesprin-4 expression is low or absent. We also observed this expression correlation in a panel of breast cancer cell lines: lines that model less aggressive breast cancer express nesprin-4, while lines that represent TNBC have low or no nesprin-4 expression. We found that this expression trend is unique for nesprin-4, as we did not observe a similar trend with either Nesprins 1, 2, or 3.

To understand if nesprin-4 expression impacts patient outcome, we analyzed the publicly available survival data. We found that nesprin-4 expression offers a clear advantage for metastasis-free survival. Patients with high nesprin-4 expression have a statistically greater metastasis-free survival at 5 years than patients with low nesprin-4 expression. However, this correlation is lost by 10 years, and when we examined the overall survival, elevated nesprin-4 expression trended with a decreased overall survival.

We used breast cancer cell lines to model nesprin-4 expression changes and understand how nesprin-4 impacts breast cancer cells. MCF7 and T47D (luminal A, nesprin-4-positive) and MDA-MB-231 (TNBC, nesprin-4-negative) cells were subjected to nesprin-4 knockdown or overexpression, respectively. We found that nesprin-4 promotes cell polarization and makes cells more efficient at 2D and 3D migration. Surprisingly, even the highly motile and invasive cell line MDA-MB-231 became more invasive when nesprin-4 was expressed (Figure 4 and Figure 7). The increase in migration and invasion seen in our assays supports a role for nesprin-4 in making breast cancer cells more aggressive, which is consistent with the decreased overall survival of patients whose cancers have high nesprin-4 expression.

Migrating cells are known to establish a centrosome–nucleus axis in the direction of migration [41]. Nesprin-4 has not previously been implicated in establishing this migration-associated polarization. However, nesprin-4 is known to bind the kinesin-1 motor protein [44,45] and, by recruiting kinesin-1 to the nuclear envelope, promotes association of the nucleus with the microtubule network. The interaction between microtubules and nuclei is known to promote nuclear positioning [43,59,60,61]. Since microtubules generally bind the centrosome with their minus end, a plus-end directed motor, such as kinesin-1, at the nucleus would lead to a separation of centrosome and nucleus, which is what we observed. Nesprin-4-driven polarization has previously been observed in HeLa cells and in the sensory hair cells of the outer ear [43,44], and now our data show that nesprin-4 can also have this role in migrating breast cancer cells. Nesprin-4 drives a rear-ward nuclear localization and a centrosome localization towards the leading edge, resulting in a more efficient migration in 2D and invasion in 3D.

The actin cytoskeleton plays a key role in migration, and actin reorganization is important for directional motility (recently reviewed in [62].) We would suggest that nesprin-4-driven polarization affects the actin cytoskeleton, not through a direct interaction, as was recently shown for Nesprin-2G [63], but rather through a number of indirect interactions between microtubules and actin, including changes in Rho GTPase activity [64,65,66]. Indeed, microtubule-driven cell polarization has been shown to promote actin–myosin contractility [67].

While we showed that nesprin-4 drives a more migratory and invasive phenotype, we also found that nesprin-4 blocks intravasation in an organ-on-chip assay. Though the MDA-231-N4 cells migrated through the ECM more efficiently than the parental MDA-MB-231 cells (Figure 7) (consistent with the nesprin-4-driven increased 3D migration we showed for all cells in this study), when these cells reached the endothelial cell layer, the expression of nesprin-4 blocked intravasation. This finding aligns with the patient data: higher nesprin-4 levels are associated with longer distant metastasis-free survival. For cells that are most likely to metastasize (i.e., TNBC), nesprin-4 expression can prevent intravasation and thereby reduce metastasis. The underlying molecular mechanism for this observation is likely based in the same mechanism that allows nesprin-4 to increase migration and invasion. The nesprin-4-mediated connections between nucleus and microtubules that leads to polarization will likely also cause an overall increase in cellular and nuclear stiffness. The expression of LINC complexes is known to affect cytoplasmic and nuclear stiffness [18,27,42]. Furthermore, it is well-established that a stiff nucleus blocks migration through constricted spaces [9,11,13]. Interestingly, granulocytes are deficient in LINC complex components; these cells are soft and move relatively freely in and out of blood vessels and extracellular spaces to reach sites of infection [42]. Based on our data, one could speculate that the inhibition of intravasation in nesprin-4-positive tumors should prevent their metastasis. In other words, enhanced expression of nesprin-4 in tumors should have a protective role. Although this could be true if the expression of nesprin-4 remained stable during tumorigenesis, tumor progression and metastasis are dynamic processes associated with major changes in gene expression and epithelial-to-mesenchymal transition (EMT). Our current understanding indicates that the most invasive cells are those that have epithelial–mesenchymal plasticity (EMP), i.e., cells that can go through EMT but also revert to more epithelial phenotypes (MET) [5,68,69]. We hypothesize that nesprin-4 is a marker of EMT that turns off late in the transition, promoting efficient migration and invasion during the earlier stages of progression, turning off to allow for intravasation, and then turning back on during MET to allow for efficient colonization at the distal site. This on/off/on toggle during EMP could explain why elevated nesprin-4 levels in breast cancer can be associated with better distal free survival but, at the same time, a poorer overall survival.

## 5. Conclusions

In conclusion, we found that nesprin-4 expression varies in breast cancer, with TNBCs showing the lowest nesprin-4 expression. Nesprin-4 actively positions the nucleus and centrosome and promotes 2D and 3D migration and invasion of breast cancer cells. In TNBC, exogenous expression of nesprin-4 promotes invasion but restricts intravasation. These findings suggest a dual role for nesprin-4 in breast cancer progression, supporting directed migration and invasion, while at the same time preventing intravasation. We propose that nesprin-4 is an example of how dynamic changes in protein expression are required for cancers to effectively metastasize.

## Data Availability

The original contributions presented in this study are included in the article/supplementary material. Further inquiries can be directed to the corresponding author.

## Supplementary Materials

The following supporting figures can be downloaded at: https://www.mdpi.com/article/10.3390/cells14191484/s1. Figure S1: Expression of nesprin-1, nesprin-2, and nesprin-3 in 62 breast cancer cell lines. Figure S2: Kaplan-Meier analysis of overall survival (OS) over a 15-year follow-up period for breast cancer patients based on low (black) vs. high (red) nesprin-4 expression. Figure S3: Survival analysis of nesprin-4 expression in breast cancer patients over a 10-year follow-up period. Figure S4: RT-qPCR of nesprin-4 mRNA expression. Figure S5: Immunofluorescence microscopy of breast cancer cell lines with anti-nesprin-4 antibody. Figure S6: Effect of nesprin-4 on 2D cell migration using a linear scratch wound assay. Figure S7: Nesprin-4 increases the 3D migration of breast cancer cells. Figure S8: Nesprin-4 promotes the leading-edge localization of centrosomes.

## Abbreviations

The following abbreviations are used in this manuscript:

EMT	Epithelial-to-Mesenchymal Transition
MET	Mesenchymal-to-Epithelial Transition
EMP	Epithelial–Mesenchymal Plasticity
ECM	Extracellular Matrix
LINC	Linker of Nucleoskeleton and Cytoskeleton
TNBC	Triple-Negative Breast Cancer
N4	nesprin-4
KD	Knockdown
