# Supplementary material for: The Role of Nesprin-4 in Breast Cancer Migration and Invasion
Source: Cells. 2025 Sep 23;14(19):1484. doi: 10.3390/cells14191484 (PMC12523417; doi:10.3390/cells14191484)
Supplement: Supplementary file 1 [file cells-14-01484-s001.zip › cells-3737440-supplementary.pdf]

Supplementary Figures

A

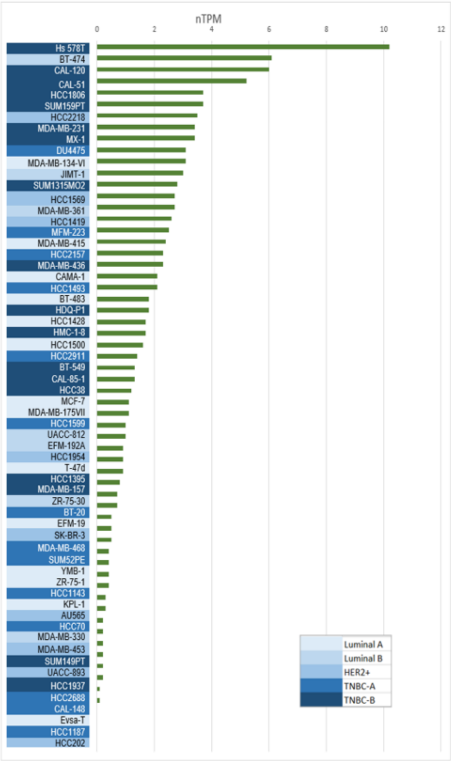

B

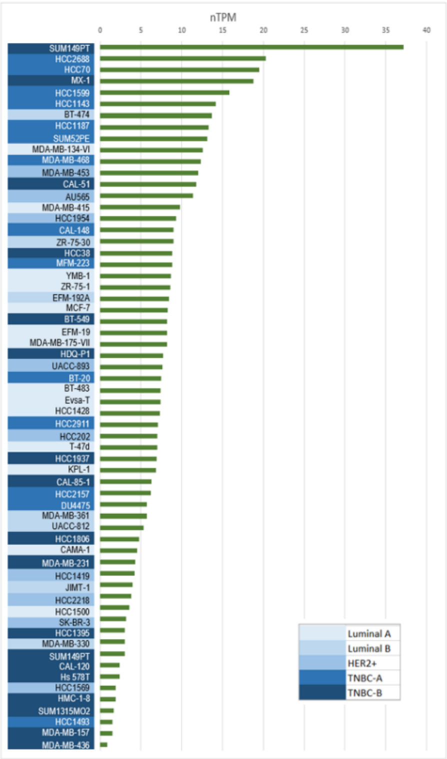

C

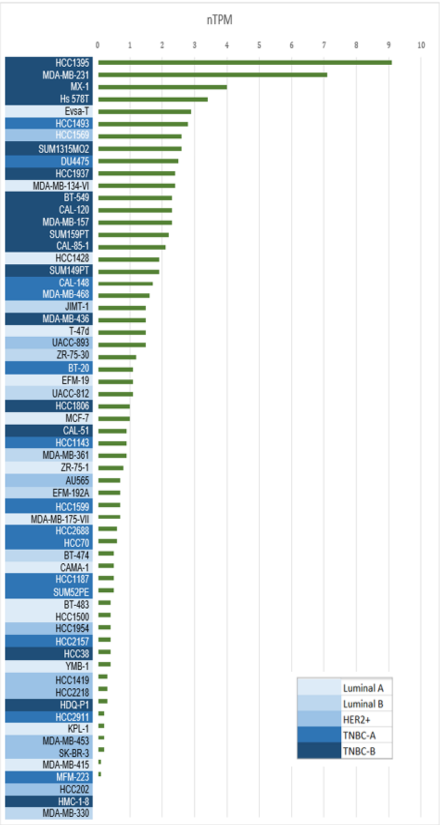

Figure S1. Expression of nesprin-1, nesprin-2, and nesprin-3 in 62 breast cancer cell lines. **(A)** Illustrates the normalized transcripts per million (nTPM) expression levels of nesprin-1 (*SYNE1*) across 62 breast cancer cell lines. **(B)** Demonstrates the nTPM expression levels of nesprin-2 (*SYNE2*) in the same 62 breast cancer cell lines. **(C)** Shows the nTPM expression levels of nesprin-3 (*SYNE3*) in 62 breast cancer cell lines. Cell lines are categorized based on their molecular subtypes: Luminal A, Luminal B, HER2+, TNBC-A, and TNBC-B, with a light-to-dark blue gradient corresponding to the degree of breast cancer subtype aggressiveness. The cell lines are ranked in descending order based on their nesprin expression levels (green bars).

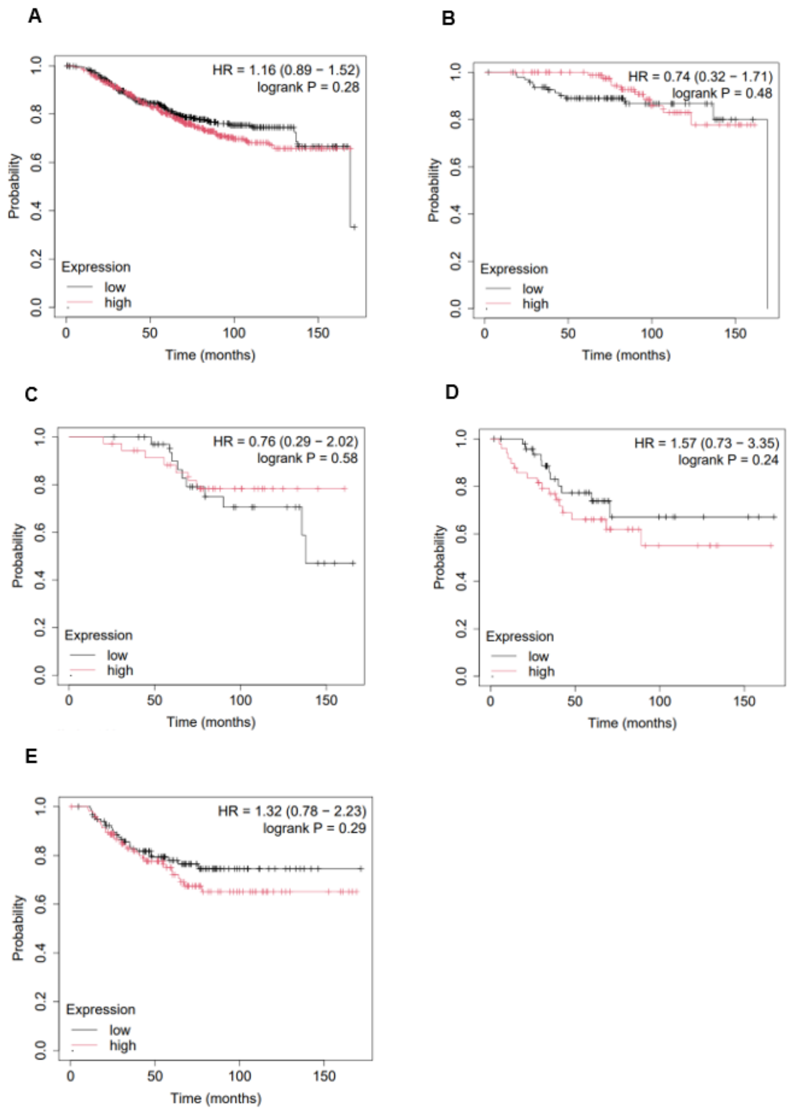

Figure S2. Kaplan-Meier analysis of overall survival (OS) over a 15-year follow-up period for breast cancer patients based on low (black) vs. high (red) nesprin-4 expression. Survival probabilities were analyzed using the log-rank test. **(A)** shows the overall survival for all the breast cancer subtypes combined (n = 1879). **(B)** Luminal A subtype (n = 194), **(C)** Luminal B subtype (n = 201), **(D)** HER2-positive subtype (n = 98), and **(E)** Triple-negative breast cancer (TNBC) subtype (n = 333). Hazard ratios (HR) with 95% confidence intervals and log-rank p-values are shown for each comparison. No statistically significant differences in overall survival were observed between high and low nesprin-4 expression groups in any subtype.

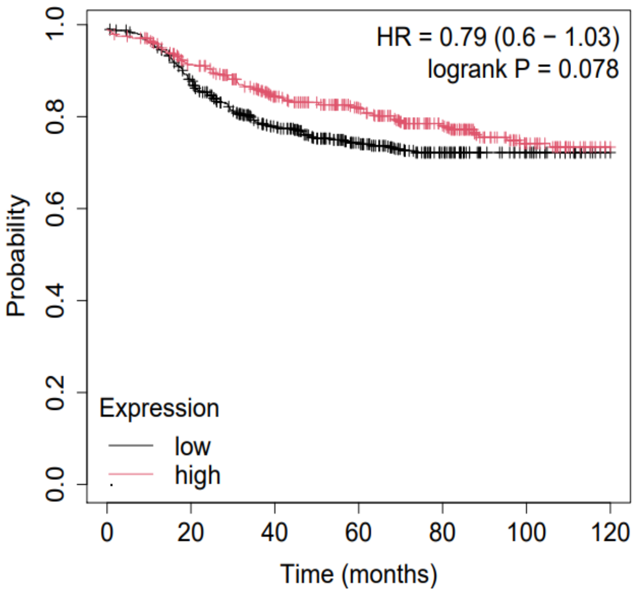

Figure S3. Survival analysis of nesprin-4 expression in breast cancer patients over a 10-year follow-up period. Kaplan-Meier plot demonstrates the probability of distant metastasis-free survival (DMFS) over time for *n* = 2765 patients with breast cancer.

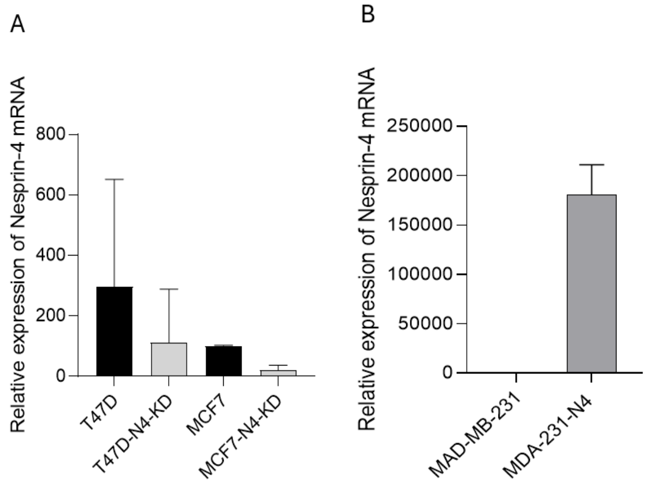

Figure S4. RT-qPCR of nesprin-4 mRNA expression. **(A)** Nesprin-4 mRNA levels are high in non-invasive breast cancer cell lines T47D and MCF7 and are reduced by nesprin-4 shRNA. **(B)** Nesprin-4 mRNA levels are not detectable in MDA-MB-231. We confirmed increased mRNA levels in MDA-MB-231 cells transfected with exogenous nesprin-4.

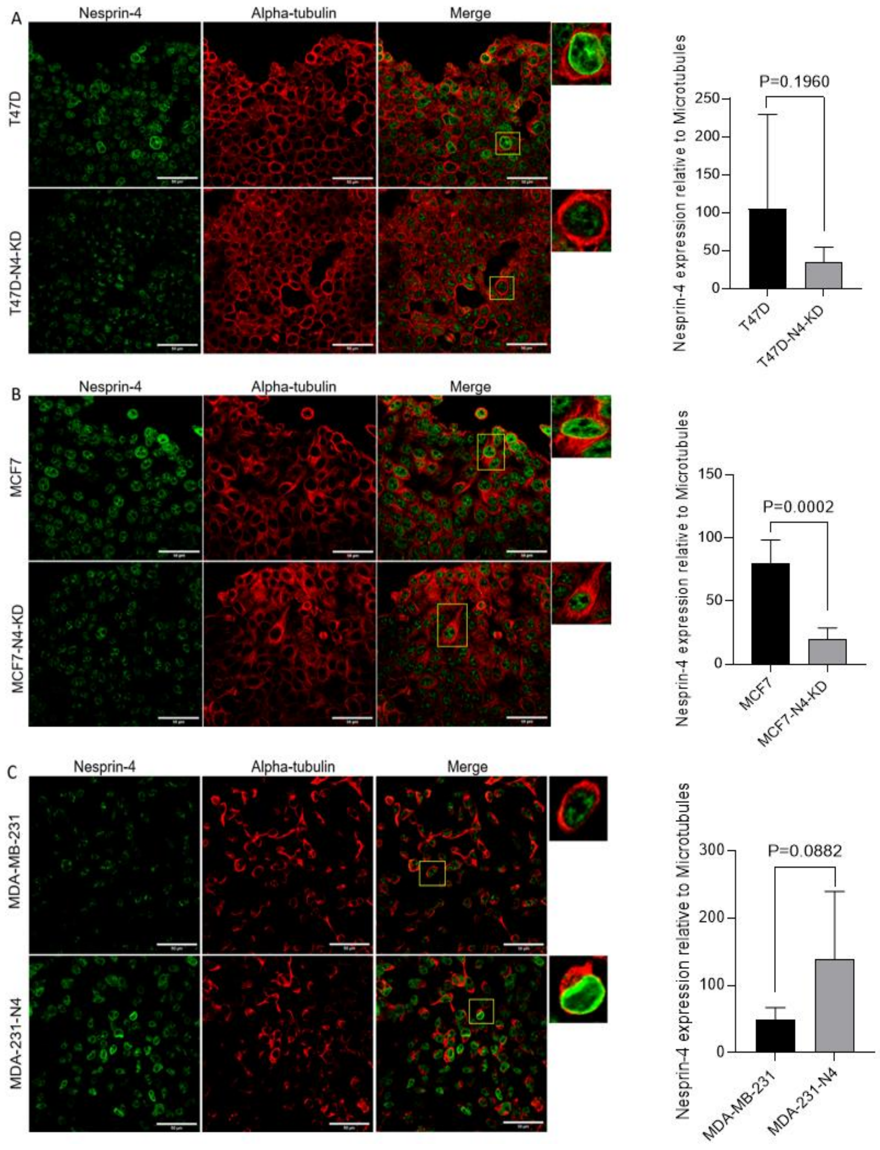

Figure S5. Immunofluorescence microscopy of breast cancer cell lines with anti-nesprin-4 antibody. Nesprin-4 was visualized via an anti-nesprin-4 antibody (Green). Microtubules were visualized using an alpha tubulin antibody (Red). The representative images show that nesprin-4 is highly expressed at the nuclear envelope in wild-type T47D and MCF7 cells and is decreased by nesprin-4 knockdown. The MDA-MB-231 cells have no detectable nesprin-4 expression. Introducing nesprin-4 into these cells results in the nuclear envelope expression of nesprin-4. The expression of nesprin-4 and tubulin was digitally quantified in ImageJ and graphed as a ratio indicating changes in nesprin-4 expression.

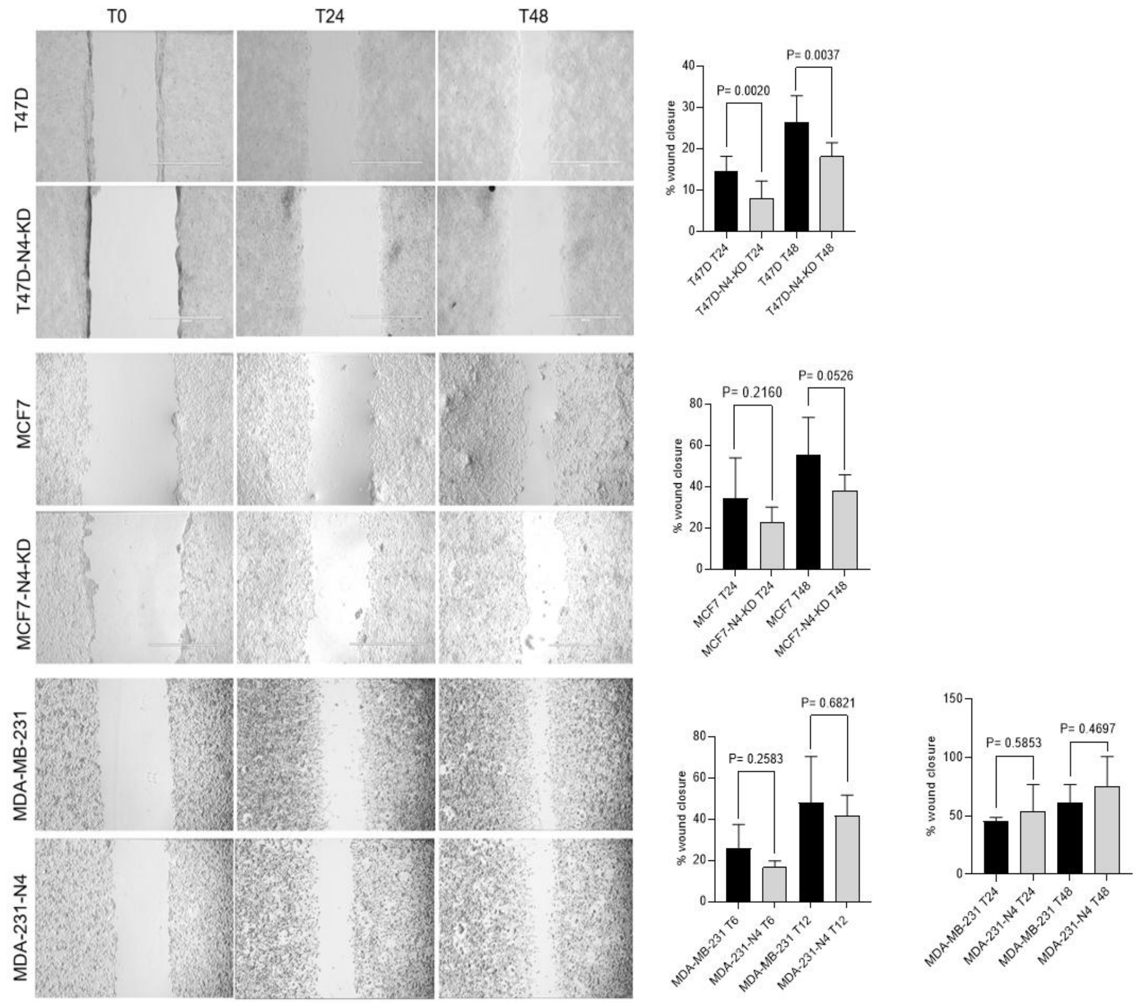

Figure S6. Effect of nesprin-4 on 2D cell migration using a linear scratch wound assay. A scratch wound healing assay was performed by making a straight-line scratch across the confluent cell monolayer. Images of the wound area were taken at 0, 24, and 48 hours at 4X magnification. Significant differences between wild-type and nesprin-4-knockdown T47D were noted at 24 hours ($P = 0.0020$) and 48 hours ($P = 0.0037$). The results for MCF7 show that nesprin-4 increases migration but at non-significant levels at 24 hours ($P = 0.2160$) and 48 hours ($P = 0.0526$). Overexpression of nesprin-4 into MDA-MB-231 does not affect cell migration at 6 hours ($P = 0.2583$), 12 hours ($P = 0.6821$), 24 hours ($P = 0.5853$), or 48 hours ($P = 0.4697$). The level of cell migration into the wound was quantified as the percentage of wound closure using the following equation: (Pre-migration area –Migration area)/ (Pre-migration area) x 100.

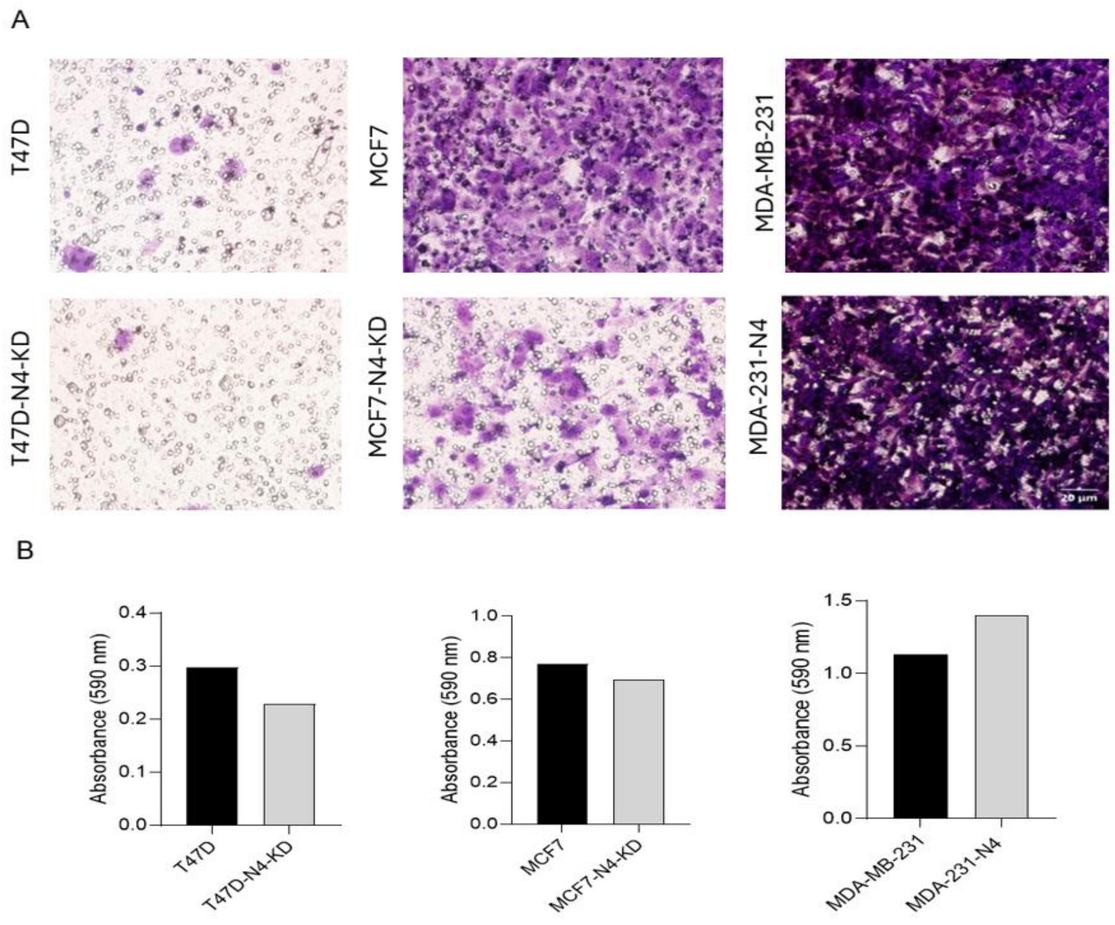

Figure S7. Nesprin-4 increases the 3D migration of breast cancer cells. 3D migration of T47D, MCF7, and MDA-MB-231 and their nesprin-4 derivative cell lines were assessed using a Transwell chamber. The cells were plated in the upper chamber with serum-free medium. The lower chamber was filled with DMEM containing 10% FBS and 50 ng/ml EGF as a chemoattractant. Non-migrating cells on the upper side of the chamber were removed, and the migrated cells on the lower side were stained with 0.2% crystal violet and imaged. Representative images of migrated cells are shown in **(A)**. The number of migrated cells was quantified **(B)** by the elution of crystal violet stain in acetic acid, and the absorbance was measured. The graphs show the values of one experiment.

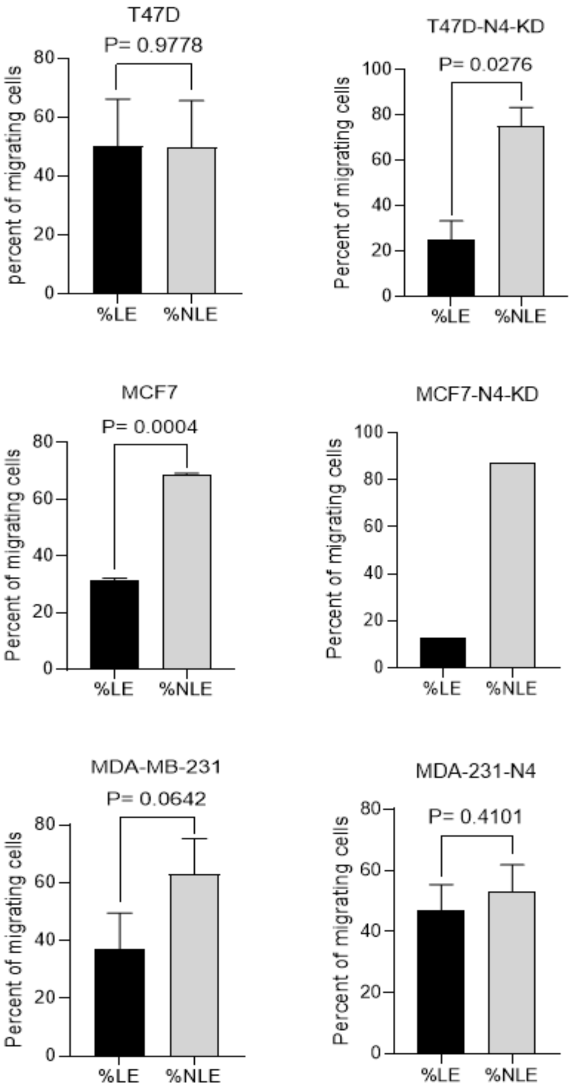

Figure S8. Nesprin-4 promotes the leading-edge localization of centrosomes. Immunofluorescent images of migrating cells shown in Figure 6 were used to evaluate the statistical differences in the percentage of migrating cells with centrosomes oriented toward the leading edge, LE (where the centrosome is positioned toward the direction of migration versus the non-leading edge, NLE (where the centrosome is positioned elsewhere, typically near the nucleus but not aligned with the direction of migration). In the wild-type T47D, there is no significant difference (*P*=0.9778) in the centrosome orientation; it shows a near-equal centrosome orientation (~50% for both LE and NLE). Knockdown of nesprin-4 in T47D increases centrosome orientation toward the NLE (~80%). It decreases orientation toward the LE (~20%). In the wild-type MCF7, ~70% of centrosomes are oriented toward NLE, with ~30% toward the LE. Knockdown of nesprin-4 enhances the NLE (~80%), reducing LE orientation (~15%). In MD-MB-231, a slight increase in NLE orientation (~70%) over LE (~35%) is observed; the difference is not statistically significant (*P*=0.0642). Overexpression of nesprin-4 in MDA-MB-231 results in a slight increase in the centrosome orientation toward LE (~45%) and a slight decrease toward the NLE (~55%), with no significant difference observed (*P*=0.4101)
